# Matters Arising: Immortal time bias in the analysis of drug prescription trajectories

**DOI:** 10.1038/s41746-022-00722-6

**Published:** 2022-12-23

**Authors:** Daniel Mølager Christensen, Gunnar Gislason, Thomas Gerds

**Affiliations:** 1 Danish Heart Foundation, Copenhagen, Denmark; 2 Department of Cardiology, Herlev and Gentofte University Hospital, Copenhagen, Denmark; 3 Department of Clinical Medicine, University of Copenhagen, Copenhagen, Denmark; 4 Department of Public Health, Section of Biostatistics, University of Copenhagen, Copenhagen, Denmark

**Keywords:** Epidemiology, Outcomes research

**arising from** Alejandro Aguayo-Orozco et al. *npj Digital Medicine* 10.1038/s41746-021-00522-4 (2021)

Aguayo-Orozco et al. [1] report survival differences in patients taking renin-angiotensin system inhibitors depending on the drug prescription trajectories identified through Danish healthcare registries. They found that changing from an initial prescription of an ACE (angiotensin-converting enzyme) inhibitor to an angiotensin receptor blocker (ARB) and vice versa conferred a survival benefit compared to no change using standard statistical tools for survival analysis. They replicated this finding in data from the UK Biobank and concluded that their results may be important when updating treatment guidelines.

At the start of the prescription trajectory, it is not yet known whether the patient will change treatment later or not. But, in order to be able to change from first line treatment to second line treatment, the patient needs to survive long enough to do so. While the use of healthcare registry data to investigate prescription trajectories and related health outcomes is promising, caution must be issued when comparing treatment groups that are dynamic over time. As a general rule, one should not analyze data conditionally on what happens in the future and hence conditionally on survival [2]. In the analysis of Aguayo-Orozco et al. (c.f., Fig. 4), this causes a problem commonly known as immortal time bias [3–5]. It occurs whenever an exposure or treatment group is defined conditionally on what happens after time zero, causing a period of immortal time for the members of this group. This bias naturally leads to favorable looking survival estimates for the treatment group with the longest immortal period [4]. The immortal time bias in the analyses presented in the study by Aguayo-Orozco et al. [1] concerns the treatment groups “ACE change to ARB” and “ARB change to ACE” because they include immortal time between the first line treatment and the second line treatment.

From a clinical perspective the conclusions of Aguayo-Orozco et al., regarding the effect of the treatment trajectories on survival are also surprising, as physicians typically change or intensify antihypertensive treatment in patients who did not achieve the desired blood pressure targets. Thus, we expect that patients who switch from first line to second line treatment have more resistant hypertension and hence have lower survival chances compared to patients who stay on first line treatment. Finally, the magnitude of treatment effects reported in the study seems implausible considering the evidence from randomized clinical trials on this subject.

Observational comparative effectiveness research is fraught with methodological pitfalls, as exemplified by the present case. Data from sources such as the Danish registries are useful to monitor the real-world implementation of evidence from randomized trials and for triangulation of evidence in the absence of randomized data [6–8]. Although, it is still widely agreed that randomized clinical trials remain the gold standard for informing clinical treatment guidelines [9]. Nevertheless, methods have been proposed to emulate a target trial using observational data [10]. Patients can be randomized to well-defined treatment regimens, even if they are dynamic. In the present case, one such regimen could assign patients to initiate treatment with ACE and then change after one year to ARB, and patients who die before the change to ARB would then still count in that group. However, the appropriate statistical analysis of the effects of dynamic treatment regimens in observational data requires the complex statistical tools of modern causal inference [8, 11]. Additionally, an important assumption of this method is that the treatment effects are identifiable from the data and not hidden behind unobserved confounding.

In summary, we agree with the authors that the analysis of observational data is valuable in describing patient trajectories and treatment patterns. However, the evaluation on treatment effects should always be based on sound clinical judgement and solid statistical methodology.

## Data Availability

Data sharing not applicable to this article, as no data were generated or analyzed in the making of this article.
